# The research progress on periodontitis by the National Natural Science Foundation of China

**DOI:** 10.1038/s41368-025-00371-x

**Published:** 2025-06-03

**Authors:** Liang Xie, Qian Chen, Hao Xu, Cui Li, Jiayu Lu, Yuangui Zhu

**Affiliations:** 1 State Key Laboratory of Oral Diseases & National Center for Stomatology & National Clinical Research Center for Oral Diseases & Research Unit of Oral Carcinogenesis and Management & Chinese Academy of Medical Sciences & West China Hospital of Stomatology, Sichuan University, Chengdu, China; 2 Department of Health Sciences, National Natural Science Foundation of China, Beijing, China; 3 The Affiliated Hospital of Stomatology, School of Stomatology, Zhejiang University School of Medicine, Zhejiang Provincial Clinical Research Center for Oral Diseases, Key Laboratory of Oral Biomedical Research of Zhejiang Province, Cancer Center of Zhejiang University, Hangzhou, China

**Keywords:** Periodontitis, Gingivitis

## Abstract

Periodontitis has emerged as one of the most critical oral diseases, and research on this condition holds great importance for the advancement of stomatology. As the most authoritative national scientific research funding institution in China, the National Natural Science Foundation of China (NSFC) has played a pivotal role in driving the progress of periodontal science by supporting research on periodontitis. This article provides a comprehensive review of the research and development progress related to periodontitis in China from 2014 to 2023, highlighting the significant contributions of the NSFC to this field. We have summarized the detailed funding information from the NSFC, including the number of applicant codes, funded programs and the distribution of funded scholars. These data illustrate the efforts of the NSFC in cultivating young scientists and building research groups to address key challenges in national scientific research. This study offers an overview of the current hot topics, recent breakthroughs and future research prospects related to periodontitis in China.

## Introduction

Periodontitis, a chronic inflammatory disease, is a destructive disease caused by dental plaque affecting the supporting tissues surrounding teeth. It is a common oral disease and the paramount reason for the loosening of teeth in adults, which causes functional disorders and affects appearance.^1^ In addition, periodontal disease status is closely associated with the risk of developing a variety of systemic diseases.^2,3^ At present, periodontitis is one of the sixth most common diseases in terms of global disease burden. Approximately 1 billion people suffer from severe periodontal diseases, accounting for 19% of adults.^4^

## Epidemiology and impact of periodontitis

The prevalence of severe periodontitis significantly increased to 8.44% between 1990 and 2019, with approximately 1.1 billion patients diagnosed worldwide, which is considered a public health concern.^4^ In China, nearly 90% of adults suffer from varying degrees of periodontal disease.^5^ From 1990–2019, the prevalence of periodontal disease in adults increased significantly (886.61 cases per 100 000 person-years to 14738.1 cases per 100 000 person-years) in China. The disability-adjusted life-year (DALY) rate has also substantially increased by 38.2 cases per 100 000 person-years.^6^ According to data from the 2009–2014 National Health and Nutrition Examination Survey (NHANES), in the United States of America (USA), periodontitis affects 42.2% of adults aged 35–70 years, 7.8% of whom suffer from severe periodontitis.^7^ The prevalence of periodontitis is estimated to range from 41.2% to 69.0% in Portugal, which is mainly urban, making it the tenth largest affected population in Europe in 2021.^8^

Periodontitis could give rise to masticatory dysfunction and a dissatisfactory appearance. The continuous aggravation of periodontitis will eventually cause teeth to fall off.^9^ The severity of periodontal disease is reflected to an extent by the loss of teeth. According to the Fourth National Oral Health Survey of China, which is based on the latest classification standards of periodontitis, the mean number of missing teeth was 3.76 (0.72 for the 35–44 age group, 3.60 for the 55–64 age group, and 6.96 for the 65–74 age group).^5,10^

Severe periodontal disease is a middle-aged disease that has the highest incidence at age 60.^11^ According to the 2019 Global Burden of Disease (GBD) study, with increasing longevity, the DALYs substantially increased by 75% for individuals with oral disease, especially severe periodontal disease, compared with those with dental caries from 1999–2019. According to stratified statistics of different age groups, only 1.7% of younger populations were affected.^12^ With increasing age, the prevalence rate sharply increased from people aged 30–40 years and peaked in individuals aged 65–74 years.^13,14^ A higher periodontitis prevalence exists among the population aged 65–74 years than among the population aged 35–44 years in European countries.^14^ The fourth national oral health epidemiological survey report revealed that the periodontal health rates of 35–44-, 55–64-, and 65–74-year-olds in 2015 were almost 9.1%, 5.0%, and 9.3%, respectively.^5,10^ In France, the prevalence of periodontitis significantly increased from 11.4% (individuals aged 35–64 years) to 18.2% (individuals aged over 65 years). In the UK, the prevalence of periodontitis significantly increased from 10.7% (individuals aged 35–64 years) to 47.2% (individuals aged over 65 years). The highest prevalence rates in Germany ranged from 16.7% (individuals aged 35–64 years) to 57.8% (individuals aged over 65 years). With the trend of population aging occurring worldwide, oral health has received increasing attention, as it is related to quality of life. More than 30% of elderly individuals have lost at least five teeth, and almost 5% of elderly individuals have no teeth.^5,10^ China has an aging society. Severe periodontitis may damage the health of elderly individuals, and the loss of teeth caused by periodontal disease affects their chewing ability and quality of life. The United Nations proposed the “Decade of Healthy Aging (2021–2030)” Initiative. There is a consensus that oral health is one of the key indicators of the overall health of older persons.^15,16^ With the aging of the population, the social burden of periodontitis has increased rapidly. Therefore, reducing periodontitis requires close attention to oral problems in elderly individuals.

By investigating the global regional distribution ratio of patients with periodontitis, we found that economic income has an important effect on the development of periodontitis. A prospective study revealed that more than 90% of villagers without access to dental care had periodontitis. With increasing age, the probability of stage III/IV periodontitis increases.^17^ In Spain and Italy, most periodontitis treatment is paid for by self-paying or privately insured patients. Therefore, periodontal treatment is almost unaffordable for low-income families, leading to a high prevalence of periodontitis. Research has indicated that there is a direct and proportional relationship between different measurement standards, such as income, education and social classes, and the severity of periodontal diseases. Studies have shown that the average number of teeth with lower socioeconomic status, education level, or other adverse factors is lower than that with higher socioeconomic and educational indicators, impacting speech, diet, social interaction and self-esteem and even further exacerbating adverse effects and inequality. A lack of teeth will also have a negative impact on aesthetics and sharply reduce the prospect of employment opportunities. The possibility of severe periodontitis in patients with low income is 1.8 times greater than that of high-income periodontitis.^18^

Research has revealed a significant association between education level and the risk of developing periodontitis in a large population-based sample aged 45 to 74 years from northern Germany in Hamburg City.^19^ Certainly, a limitation of this research also apparently exists, which excluded relatively poor or nonnative German-speaking individuals. Data from the NHANES III, which included 13 665 participants, revealed that the prevalence of periodontitis was significantly higher in individuals with low education levels than in those with high education levels.^19^ Socioeconomic status has been widely recognized as one of the risk factors for the development of periodontitis.^20,21^ An observational study revealed that socioeconomic status was negatively associated with tooth loss and directly related to worsening periodontal status in 118 Italian adults over fifty years of age.^22^ A cross-sectional or epidemiological study of a representative sample of the Spanish adult population also demonstrated similar relevance between socioeconomic factors.^23^

The goal of the global tobacco and alcohol industries is to pay more attention to emerging economies or particularly vulnerable population groups in low- and middle-income countries.^24^ Studies have shown that the growth rates of sugar consumption, tobacco use and alcohol use, which are risk factors for periodontitis, are markedly higher in low- and middle-income countries than in high-income countries. Poor oral hygiene is the main risk factor for periodontitis. A systematic review including 15 studies revealed a positive relationship between smoking status and the risk of developing periodontitis, which increased the incidence of periodontitis by 85%.^25^ Calculation of the “population attributable risk fraction” revealed that quitting smoking reduced the risk of periodontitis by approximately 14% in a prospective longitudinal study.^26^ In China, periodontitis is more severe in smokers than in nonsmokers. However, there was no significant difference in the severity of periodontitis between men and women.^5,10^ Therefore, the focus should be on assessing the potential dose-dependent effect of cigarette smoking on periodontitis in future prospective longitudinal research.

Periodontal health is a crucial component of general health, exhibiting a synergistic or bidirectional relationship. In recent decades, many studies have shown potential relationships between periodontal disease status and the risk of developing many other chronic diseases, including evidence of a correlation between periodontal disease status and the risk of systemic diseases, such as diabetes, cardiovascular disease and chronic obstructive pulmonary disease (COPD). Periodontitis is considered the sixth complication of diabetes mellitus (DM) and is closely related to blood glucose levels.^27^ Compared with the periodontally healthy population, patients with periodontitis have higher levels of blood glucose, indicating a greater incidence of diabetes.^28^ A cohort study revealed that type 2 DM (T2DM) could increase the risk of periodontitis by 34%.^29^ A large-scale epidemiological study revealed that the incidence of periodontitis without diabetes was 10.6%, whereas 70.6% of patients with diabetes had moderate periodontitis, and 28.5% had severe periodontitis.^30^ Therefore, effective oral hygiene maintenance and periodontal support are essential for the control of diabetes. In addition, epidemiological studies have revealed a positive correlation between periodontitis and cardiovascular disease. In one cohort study in 2018, the risk of cardioembolic and thrombotic stroke in patients with periodontitis was double that in periodontally healthy individuals.^31^ A case-in-comparison of human epidemiological data revealed that the incidence of periodic inflammation in patients with myocardial infarction was significantly increased in 805 patients with myocardial infarction. The risk of developing myocardial infarction in patients with medium and severe periodontitis is significantly elevated accordingly.^32^ In conclusion, periodontal disease status is closely related to systemic disease risk. Therefore, research on periodontitis will be helpful for maintaining human health and improving quality of life. Life-cycle epidemiological studies are also increasingly important for disease prevention and risk factor assessment. A randomized controlled trial suggested that supplementation with vitamin D could enhance the curative effect on periodontitis.^33^ An analysis of NHANES data revealed that exposure to polycyclic aromatic hydrocarbons (PAHs) is positively correlated with the risk of developing periodontitis, which may provide new insights into the prevention of periodontitis from the perspective of environmental exposure.^34^ Similar results also exist between mixed aldehyde exposure and the risk of developing periodontitis.^35^

Epidemiological studies have suggested that periodontal disease has harmful effects locally and systemically. Reducing risk is very important in the treatment process. Successful risk management is necessary for effective long-term treatment. It needs to be emphasized that risk is not necessarily related only to patient factors, and increasing the understanding of risk factors for the development of periodontal disease will help us attain a more comprehensive understanding of periodontal disease.^20^ Through a retrospective analysis of the database, researchers reported that a high prevalence of gingivitis exists in Chinese adolescents; thus, improving the awareness and prevention of periodontal disease in adolescents is necessary.^36^ Noteworthy, the level of knowledge of oral health among Chinese people is unbalanced, and the incidence of periodontal disease is still relatively high. Therefore, it is necessary to strengthen age-specific hygiene guidance and synergize prevention and treatment to promote oral health.^37^ By comprehensively identifying risk factors, such as lifestyle, the interaction of diseases, and the burden of aging, we can gradually increase awareness of periodontitis disease. This evidence highlights the urgent need for increased awareness of the burden of periodontitis, improved prevention policies and increased access to care. However, the therapeutic regimen is limited to basic treatment and the use of drugs, which are maintained for many years. These therapies cannot reverse and cure the disease, and the effect is maintained for a short time so that patients require permanent treatment to maintain health care. Therefore, the need to identify specific targets and new strategies for treatment is urgent. China’s scientific research support system is gaining proactive momentum. As an important supporting department of national scientific basic research, the Natural Science Foundation of China (NSFC) strongly supports scientific innovation and encourages scholars to constantly innovate and develop new therapeutic concepts in periodontitis.

## Role of the NSFC in promoting development in the field of periodontitis research in China

The National NSFC was approved by the state council in 1986, which is under the management of the Ministry of Science and Technology of China. The NSFC supports basic research, adheres to free exploration, and plays a guiding role, in accordance with national development science and technology policies and plans in China. The main tasks of the NSFC include three major goals: implementing source innovation, providing scientific and technological talent, and enhancing the innovation environment. The formulation and implementation of discipline development strategies promote the balanced and coordinated development of disciplines. The NSFC cultivates talent with innovative thinking and abilities to provide basic research conditions and optimize the research environment. To match the major scientific topics in this discipline and national strategies, the NSFC promotes a series of basic, strategic, and forward-looking priority research fields.

The NSFC has established corresponding project types based on the development trends of science and technology and national strategic needs. After continuous optimization and adjustment, a reasonable and complete funding system has been formed, which contains eighteen types of projects. The project supports scientific and technical personnel engaged in basic research to select topics, carry out innovative scientific research, and promote the balanced, coordinated and sustainable development of various disciplines. Key projects conduct in-depth and systematic innovative research on existing research directions or disciplinary growth points, promote the development of disciplines, and facilitate breakthroughs in several important areas. The major projects are facing major scientific issues at the forefront of science and the development of the national economy, scientific and technological development, society and national security. These projects give full play to leading roles and enhance basic research sources via overall deployment, multidisciplinary cross-research and comprehensive research. The international cooperation research project funds scientific and technical personnel based on the international science forefront, effectively utilizes international scientific and technological resources, and carries out substantial international cooperation research in the principles of equal cooperation, mutual benefit, and results sharing to improve China’s scientific research level and international competitiveness. The Youth Scientists Fund project supports young science and technology personnel in choosing topics within the scope of funding of the Science Fund, carrying out basic research and cultivating the ability to independently host scientific research projects that involve innovative research conducted by young science and technology personnel and inspire them to begin thinking about basic research development. The National Outstanding Youth Science Fund project supports young scholars who have achieved outstanding achievements in basic research to carry out innovative research, promote the growth of young scientific and technological talent, attract overseas talent, and cultivate and create a group of excellent academics at the forefront as world technology leaders.^38^

### Funding for long-term periodontitis research

**Fig. 1:**
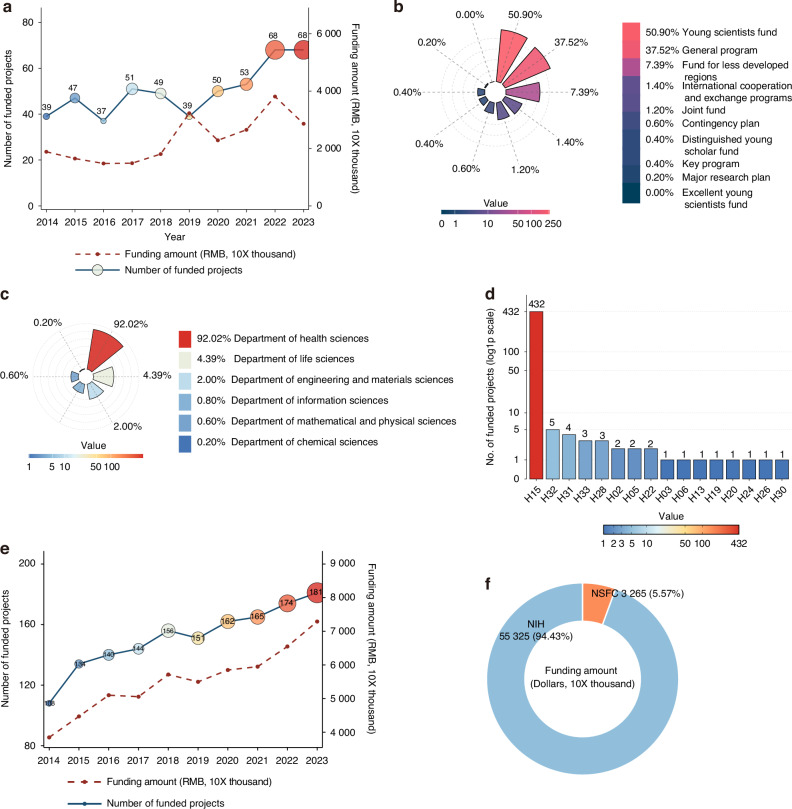
Statistics the funding of NSFC in the periodontitis related field in China from 2014 to 2023. **a** The number of funded projects and the funding amounts annually. **b** The proportion of different funded project types in the periodontitis related field in NSFC. **c** The proportion of different funded departments in the periodontitis related field in NSFC. **d** The number of different funded primary application codes in the periodontitis related field in NSFC. **e** The number of funded projects by NIH annually. **f** Comparison of the amounts of projects funded by the United States and China

During the past decade (2014–2023), the NSFC has funded abundant periodontitis-related studies, totaling 501 projects and a cumulative amount of approximately 231.60 million Ren Min Bi (RMB). As shown in Fig. 1a, the number of funded projects increased annually. In 2023, the number of funded projects was almost equivalent to twice as many projects as in 2014. The trend of the number of funded projects was basically increasing. One major project was funded in 2019, and one distinguished young scholar was funded in 2016 and one in 2020. There were drastic increases in funding amounts in 2019 and 2022. The imbalance between the sudden increase in the amount of funding and the decrease in the number of funded projects was due to a major project that was funded in 2019. Similarly, funding for international cooperation and exchange programs and joint projects was responsible for the surge in funding amount in 2022 (Fig. 1a).

Among the periodontitis-related projects funded in recent decades, projects headed by young scientists accounted for more than half (255 projects, 50.90%), which greatly helped young scientists invest in scientific research and promoted innovations and breakthroughs. Second, the number of general programs funded accounted for 37.52% (188 projects), which supported researchers in choosing interesting research topics for innovative scientific research. In addition, 37 projects (7.51%) were funded in less economically developed provinces to encourage scientific research and development in these areas. The key projects and major research plan funded 3 projects, accounting for approximately 26.4% of the funding amount, which supported leaders in creating comprehensive and systematic development of the periodontitis field to solve key problems in periodontitis-related research fields (Fig. 1b).

Six departments participated in periodontitis research. The largest department funded for periodontitis research in the NSFC is the Department of Health Sciences, which has 461 projects (92.02%) (Fig. 1c). Fewer projects were funded by the other five departments, which represented the breadth and correlation of the studies on periodontitis. Half of the primary application codes in the Health Sciences Department are involved in the study of periodontitis (16 of 32), indicating that periodontitis is closely associated with numerous areas of medical science (Fig. 1d). Oral craniomaxillofacial science, code H15, is the main application code used to fund the periodontitis field (432 projects). The science of traditional Chinese medicine is the second largest application code in the field of periodontitis (5 projects). Traditional Chinese medicine is the most distinctive research direction in China, and its funding in the field of periodontitis will contribute to the discovery of unique periodontitis treatments in the future.

Compared with other predominant countries, the National Institutes of Health (NIH) of the USA funded 1 515 projects (5 532.25 million USD), with a gradual and steadily increasing trend in periodontitis-related fields from 2014 to –2023, which is far greater than the total funding and amount in China (Fig. 1e, f). The investment in the field of periodontitis in China is far less than that in the USA.

### Funding for research scientists in China

**Fig. 2:**
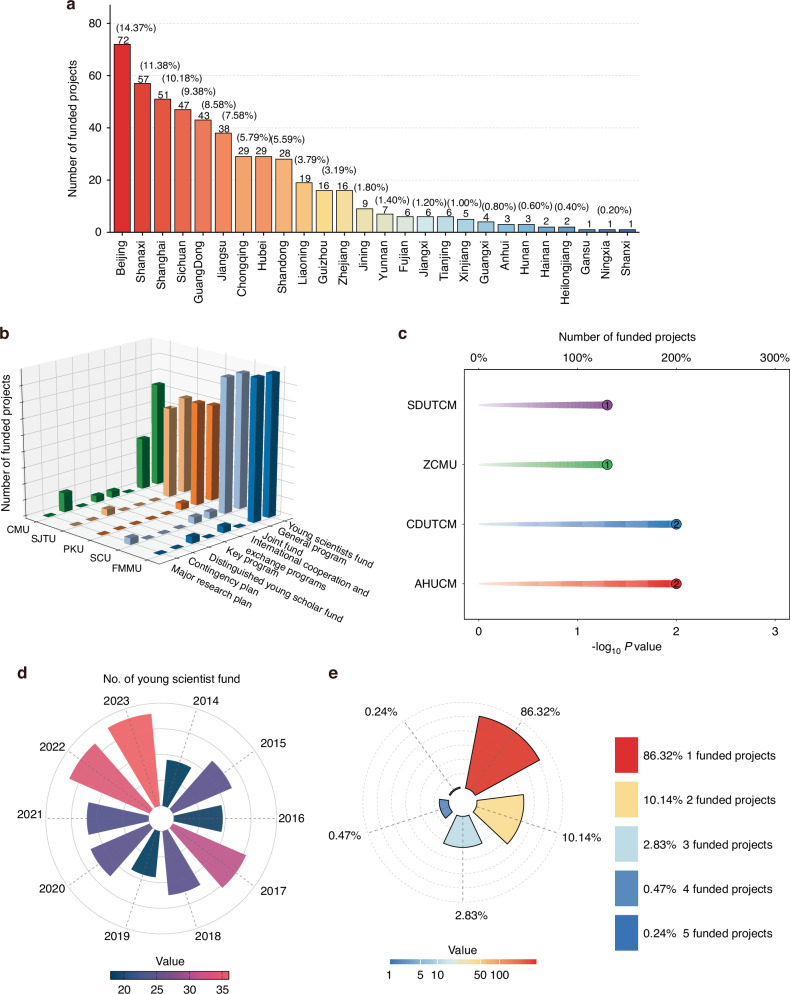
Statistics on the number of projects NSFC funded in the distribution of universities and provinces in China from 2014 to 2023. **a** The distribution of funded projects in different provinces of China in the periodontitis related field. **b** The distribution of different funded projects in the top five NSFC supported universities in the periodontitis related field. **c** The distribution of different funded projects in the top four NSFC supported traditional Chinese medicine universities in the periodontitis related field. Anhui University of Chinese Medicine (AHUCM); Chengdu University of Traditional Chinese Medicine (CDUTCM); Zhejiang Chinese Medical University (ZCMU); Shandong University of Traditional Chinese Medicine (SDUTCM). **d** The number of young scientists funded projects in the periodontitis related field annually. **e** Statistics of the number of supported scholars in the periodontitis related field

With the increasing standard of living for residents of China, their attention to oral hygiene has increased. The number of NSFC-funded periodontitis-related projects has likewise steadily increased. As of 2023, the number of projects funded was double that in 2014 (Fig. 1a). The statistics of funding projects related to periodontitis in the past 10 years revealed that 69 university institutions have invested in periodontitis-related projects. Research on periodontitis has been conducted in the majority of provinces in China, and most of the involved institutions are concentrated in developed regions. The number of funded projects in economically developed provinces (Beijing, Guangdong, Shanghai and Jiangsu) accounted for approximately 40.5%. This result also confirms that a lower prevalence of periodontitis exists in economically developed areas, which is related to understanding oral health and overcoming periodontitis disease. In comparison, there were only 3 funded projects in economically underdeveloped provinces (Ningxia, Shanxi and Gansu) (Fig. 2a). The projects supporting less developed regions have been partially successful, but a small number of regions still need further support to further mitigate the differences in periodontitis investment between different economic regions in China.

According to the analysis of the proportion of top dental medicine research universities in the strategic development plans, the top five universities in periodontitis-related projects assisted by the NSFC are The Fourth Military Medical University (FMMU, 46 projects, Shaanxi), Sichuan University (SCU, 45 projects, Sichuan), Peking University (PKU, 32 projects, Beijing), Shanghai Jiao Tong University (SJTU, 30 projects, Shanghai) and Capital Medical University (CMU, 29 projects, Beijing). Young scientist funds and general programs account for the largest proportions of funding in these universities. In addition, these universities are supported by major or key projects, which are crucial for the growth of the core team (Fig. 2b). These universities have high-level experimental platforms, and more researchers are leading periodontitis research to further levels.

As shown in Fig. 2c, the application code for traditional Chinese medicine is second in the field of periodontitis research, and as a traditional and unique field, it has an essential role in periodontitis research. There are four Chinese medical universities funded with 6 periodontitis-related projects distributed in Anhui, Zhejiang, Chengdu and Shandong Provinces (Fig. 2c). Traditional Chinese medicine may become a new domain in the treatment of periodontitis.

### Strengthening the construction of scientific research groups

Continuous progress in the periodontitis-related field has not been achieved overnight. The echelon construction of the scientific research team has aided in the continuous development of periodic research. A research group includes a principal investigator, formal researchers and graduate students. With the continuous development of periodontitis-related research systems, many graduate students will become formal researchers leading the development of periodontitis research in the future. To date, NSFC-funded projects have formed an echelon project funding model at some universities, such as the Fourth Military Medical University and Sichuan University. Because of NSFC series funding, research on periodontitis is developing continuously. Moreover, the NSFC has funded different kinds of projects, which have led to the formulation of talent groups conducive to the development of periodontal field research.

The support of young scientists provided by the NSFC is the driving force for the development of periodontitis-related research. Young researchers have expanded their research ideas and conducted in-depth research through the original obtained funding. As presented in Fig. 2d, the number of NSFC-funded youth scientist programs doubled from 2014 to –2023. This rapid growth rate also indicates the rapid development of research in periodontitis-related fields in China. There were 366 researchers who had previously received NSFC-funded projects (86.32%). A total of 366 researchers had previously received NSFC-funded projects (86.32%). Due to the foundation of youth scientist programs, young researchers also have the opportunity to acquire general programs. Approximately 10.14% of researchers have received two NSFC-funded awards for projects. Twelve researchers received three NSFC awards (2.83%). Only one scholar obtained 5 rounds of funding (0.24%) (Fig. 2e). These data indicate that multiple grants from the NSFC could help scholars gain breakthrough research results in the periodontitis field. Thus, NSFC funding dedicated to young scholars is the basis for achieving major research breakthroughs in the future.

Considering the importance of the oral health of the whole population, combining national needs and grasping the forefront of world science, the NSFC supports scholars in conducting key periodontitis-related projects exploring innovative research topics. To date, there are three key and major project areas funded by the NSFC in the periodontitis field: the mechanism of periodontal homeostasis, periodontal regeneration and periodontal medicine. The funds promote in-depth, systematic and innovative research on well-founded research directions or disciplinary growth points to promote breakthroughs in several important fields or scientific frontiers. The funded researchers have become academic leaders and top scientists in periodontitis research in China.

## Research progress of periodontitis in China

From 1990 to 2019, the incidence, prevalence and DALY rates of periodontal disease presented an overall upward trend in China. In 2019, the incidence of periodontal disease was 1 269.9 per 100 000 people, the prevalence rate was as high as 14 738.1 per 100 000 people, and the DALY rate was 96.3 per 100 000 people, which was significantly higher than that reported in 1990 in China.^6^ The occurrence and development of periodontal disease are closely related to economic status, the environment, smoking and other factors. However, adult tobacco use is more serious, which may be related to the increase in the incidence of periodontal disease in China.^5,39^ According to the age‑period‑cohort model forecast, the incidence of periodontal disease will continue to increase from 2020 to 2044, and the growth rate for women is higher than that for men in China.^6^ Healthy life begins with the teeth; oral health status reflects quality of life and health, and healthy teeth are the gateway to prevent and control systemic diseases. Oral health is a symbol marker at the civilization level and is an essential foundation for systemic health. As great importance is attached to the oral health of people at the national level, in the “Healthy China 2030” plan, oral health has been included as an important aspect of national health for the first time. To implement the outline of the “Healthy China 2030” Plan and the “Medium- and Long-Term Plan for the Prevention and Treatment of Chronic Diseases in China (2017–2025)”, the NHC has further promoted the “Three Reduction and Three Health” healthy oral action, combined with the current oral health status of Chinese residents and oral health work. The plans have proposed the following action goals: by 2025, a healthy oral social supportive environment will be formed, the oral health literacy level of the population and the formation rate of health behaviors will be greatly improved, and oral health services will cover the whole population and the whole life cycle to better meet the health needs of the people.^40^ The report of the 20th National Congress of the Communist Party of China noted that we should promote the construction of a healthy China. The Healthy Oral Action Plan (2019–2025) clearly requires adults to brush their teeth twice a day at a rate of 45%, and elderly individuals aged 65–74 should have at least 24 teeth. These goals emphasize that periodontal health is an important basis for general health. The key to the treatment of periodontitis is removing plaque and calculus and improving the living environment for the teeth. The development of good oral health habits is the most basic but crucial way to prevent periodontitis.^41^

### Publication trends in global periodontitis research

Articles are among the vital achievements of NSFC funding. A search for periodontitis-related publications in Web of Science from 2014 to 2023 revealed that the number of periodontitis-related publications increased significantly during that period. In 2014 or 2023, the countries with the highest number of periodontitis-related publications were the USA, China, Brazil, Japan and India. In 2014, the number of publications in China was only second in the world (154 publications). By 2023, China surpassed the USA in becoming the first country in the world in publication number (701 publications). The number of publications in China has dramatically increased by approximately 4-fold, with the fastest growth among all countries. The number of publications in the USA has increased steadily (216 publications in 2014 and 289 publications in 2023). In terms of publication number, the USA is closely followed by India (216 publications) and Japan (137 publications) (Table 1).

**Table 1:** Top 10 nations for Periodontitis-related publications in 2014 and 2023

Rank	2014	Publications	2023	Publications
1.	USA	216	China	701
2.	China	154	USA	289
3.	Brazil	104	India	216
4.	Japan	101	Japan	137
5.	India	64	Brazil	122
6.	Germany	62	Germany	106
7.	Turkey	54	Saudi Arabia	104
8.	England	47	Italy	102
9.	South Korea	47	Turkey	101
10.	Italy	46	England	89

**Fig. 3:**
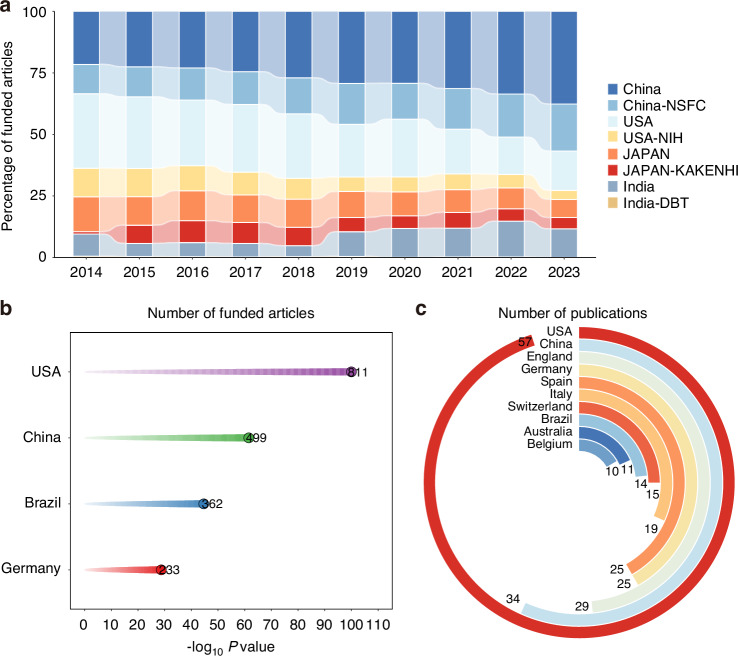
Statistics on the number of publications of China and other countries in the periodontitis related field from 2014 to 2023. **a** The percentage of publications from China, the United States, Japan, and India and funded proportions by the NSFC, the NIH, KAKENHI and DBT annually. **b** The number of Top Journals among the top four periodontitis related publications countries. **c** The distribution of highly cited publications in the top ten countries

As the most authoritative scientific research approach in China, the NSFC has made outstanding contributions to periodontitis-related publications. The NSFC has published approximately 1820 periodontitis-related publications, accounting for 52.83% of relevant publications in China during the past 10 years. With the efforts of the NSFC, the number of publications funded by the NSFC increased by 4.2 times in these 10 years (85 publications in 2014 and 355 publications in 2023), which reflects the efforts of the NSFC in China. Compared with those funded by the NIH in the USA and the national Grants-in-Aid for Scientific Research (KAKENHI) in Japan, the number of publications funded by the NIH and KAKENHI basically maintained a stable trend. Approximately 816 NIH-funded papers have been published, accounting for 32.50% of relevant publications in the USA during the past 10 years, which is similar to China. However, this ratio sharply decreased to 2.60% in the Department of Biotechnology (DBT)-funded area in India (Fig. 3a).

Articles recorded in highly influential periodontitis-related journals will gain greater credibility and value. Journals focusing on periodontitis and high-level partitions have been defined as “TOP Journals”, including the *Journal of Periodontal Research, Periodontology 2000*, the *Journal of Dental Research*, the *International Journal of Oral Science*, the *Journal of Periodontology* and the *Journal of Clinical Periodontology*. The USA contributed 811 papers in TOP Journals, accounting for 42.57%, reflecting that the influence of the USA in the periodontitis field far exceeds that in other countries. The proportions of papers contributed by researchers in China, Germany, and Brazil closely follow those of the USA, at 26.2%, 19.0% and 12.2%, respectively (Fig. 3b). With respect to information from the Web of Science, the USA published the majority of highly cited papers worldwide, accounting for 23.8% (57 papers) in 2014–2023. In terms of highly cited papers, China occupied second place, accounting for 14.23% (Fig. 3c).

### Publication trends of periodontitis in TOP Journals in China

**Fig. 4:**
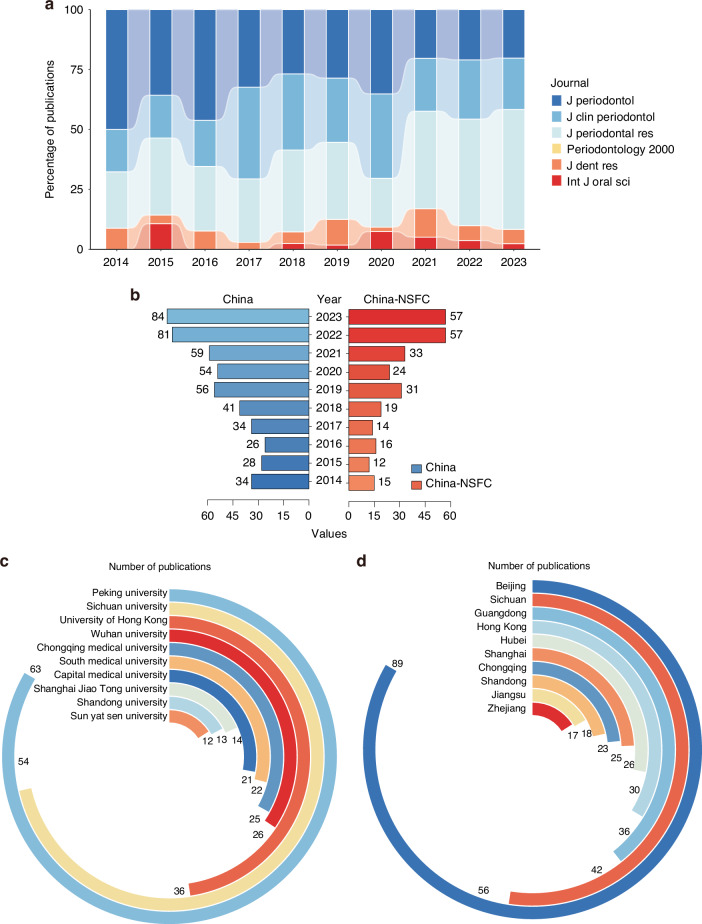
Statistics on the number of periodontitis related TOP Journals publications in China from 2014 to 2023. **a** The percentage of publications in periodontitis related TOP Journals in China annually. **b** The number of periodontitis related TOP Journals publications from China and funded proportion by NSFC annually. **c** The number of periodontitis related TOP Journals publications on the affiliation of the corresponding authors in top ten universities. **d** The distribution of top ten corresponding authors affiliation provinces in periodontitis related TOP Journals publications

In the past 10 years, the contributions of Chinese scholars in the TOP Journals related to periodontitis have had a high impact (497 publications). As shown in Fig. 4A, the number of articles published by Chinese scholars in TOP Journals on periodontitis has steadily increased annually. The majority of high-impact publications of Chinese scholars have been published in the *Journal of Periodontal Research*, the *Journal of Clinical Periodontology* and the *Journal of Periodontology*. Publications in the *Journal of Dental Research* emerged in the past three years, with approximately 5 publications per year. However, almost no articles were published by Chinese scholars in *Periodontology 2000* (Fig. 4a). Chinese researchers still need to produce high-level publications to be recognized by globally renowned scholars in the field of periodontitis. Compared with the total number of publications in TOP Journals, the proportion of NSFC-funded publications ranged from 44.12% in 2014 to 67.86% in 2023, which indicates that the influence of the NSFC in China has increased (Fig. 4b). Using that information, we statistically analyzed the number of publications of different Chinese universities in the field of top periodontitis journals and discovered that Peking University, the University of Hong Kong and Sichuan University are the top three universities with publications in TOP Journals and occupy the top positions in terms of funding by the NSFC (Fig. 4c). Among these scholars, most of the scientific research institutions were located in Beijing and Sichuan, accounting for 40.06% of top 10 institutions. The other institutions were distributed in the coastal regions(44.75%), with fewer in the central regions (Fig. 4d).

### Representative achievements in the field of periodontitis in China

Periodontitis is the sixth most common chronic disease in the world. Approximately 62.3% of adults aged over 35 years suffer from periodontitis, and the prevalence of severe periodontitis is as high as 30.6% in China.^5^ Periodontitis has become one of the most common and prominent oral health problems in China. Therefore, early prevention and control of major chronic diseases have become major challenges in the field of public health. The main goal of periodontal disease treatment is to treat infections caused by pathogenic biofilms during the periodontal period and prevent or slow the further loss of periodontal adhesion and periodontal bone loss, with the goal of preventing tooth loss. In the past decade, the NSFC has provided critical assistance to periodontitis researchers in China. Here, we summarize the representative achievements of NSFC-funded research from the following aspects.

### Periodontal pathogens

Nowadays, major deficiencies remain in the interpretation of the pathological mechanism of periodontitis, which affects new discoveries in the treatment of periodontitis and the quality of life of patients to a certain extent. Through the long-term efforts of the NSFC, researchers have conducted many studies on the pathogenesis of periodontitis and broadened our understanding of the disease. Research on the pathogenesis of periodontitis is helpful for understanding the occurrence and development of periodontitis to analyze its etiology. Periodontitis is characterized mainly by inflammatory bone resorption and connective tissue destruction caused by periodontal pathogenic infections. By collecting subgingival plaque and periodontal clinical data from patients, Yan et al. reported that the abundance levels of *Bacteroides* and *Spirochetes* were significantly greater in a periodontitis group than in a healthy population at the phylum level, whereas the abundance levels of *Actinomycetes* and *Proteobacteria* were lower in the healthy population, indicating that the dynamic changes in bacteria and their interactions are highly important to the periodontal microecology and the development of periodontitis.^42^ In clinical trials, researchers have reported that following scaling and root planning (SRP) in patients with periodontitis, the subgingival microbiota undergoes a transformation, shifting from a state of high enrichment with *Porphyromonas gingivalis* (*Pg*.), *Treponema*, and *Fretibacterium* species to a proliferation of *Actinomycetes* and *Streptococcus* species.^43^ Dynamic changes in bacteria play important roles in the development of periodontitis.^44^ Investigating etiology research helps us comprehensively understand and analyze periodontitis.

Periodontal pathogen invaded the periodontal tissue and destroyed the periodontal barrier, thus accelerating the progression of periodontitis. The expression levels of tight junction proteins in periodontitis tissue decrease, facilitating the invasion of periodontal pathogens.^45^ Neutrophils are the first immune cells in the host to respond to plaque biofilms and play dual roles in the pathogenesis of periodontitis and the host’s defense against periodontitis. In response to the immune response to periodontal pathogens, Meng et al. reported that neutrophils release web chromatin and that platelets migrate to the gingival sulcus to engulf periodontal pathogens and promote the formation of neutrophil extracellular traps (NETs).^46^ Deng et al. reported that OTUD1, a deubiquitinase that targets proteolysis, plays an important role in alleviating periodontal inflammation by restricting neutrophil activation and migration through the OTUD1–SEC23B–CD9/CD47 signaling axis.^47^ Like periodontal pathogen levels, formic acid levels decreased after nonsurgical periodontal therapy in the gingival crevicular fluid of periodontitis patients, whereas acetic acid, propionic acid and butyric acid levels increased. These findings suggest that short-chain fatty acids (SCFAs) might provide new therapeutic options for the prevention and treatment of periodontitis.^48^ Yu et al. identified *Pg*. and N1-acetylspermine as potential biomarkers of periodontitis using metagenomic sequencing and liquid chromatography‒mass spectrometry to detect microorganisms and their metabolites.^49^ Under *Pg*. stimulation, human gingival fibroblasts aggravate inflammation and ferroptosis, thus exacerbating the development of periodontitis.^50^ The researchers revealed spatial patterns of immune cells in the gums, which found that the infiltration of plasma cells significantly increased in periodontitis by using Stereo-seq.^51^ Blocking C-C motif chemokine receptor 2 (CCR2) inhibits monocyte/macrophage recruitment and effectively alleviates periodontitis.^52^ The Th1/Th2 ratio and the proportions of Th2, Th17, and Treg cells are considered closely related to the stages of periodontitis progression.^53,54^

Extensive evidence shows that *Pg*. is not only a common opportunistic pathogen in the oral cavity but also closely associated with a variety of nonoral diseases, including inflammatory bowel diseases,^55^ cardiovascular disease,^56^ Alzheimer’s disease^57^ and so on. Periodontal pathogens and the immune cascade disrupt periodontal homeostasis. The activation of the NLRP3 inflammasome and lysis of gasdermin D (GSDMD) were found in the periodontal tissues of diabetic periodontitis mice, which demonstrated the existence of pyroptosis in periodontal tissues, leading to the progression of periodontal inflammation. It was subsequently confirmed that impaired liver metabolism in patients with diabetes promoted the release of exosomes carrying FASN, leading to the pyroptosis of periodontal stem cells.^58^ In order to eliminate periodontal pathogens, researchers have designed dual crown vesicles with both antibacterial and bio-delivery functions to kill periodontal pathogens by efficiently delivering antibiotics to biofilms, thus significantly reducing plaque and periodontal inflammation.^59^ Baicalin, a flavonoid extracted from *Scutellaria baicalensis*, has been shown to have antioxidant and anti-inflammatory activities and can restore the Firmicutes/Bacteroidetes (F/B) ratio to improve alveolar bone loss in senile periodontitis model mice.^60^ The administration of baicalein promoted the osteogenesis of periodontal ligament cells (PDLCs) by activating the Wnt/β-catenin signaling pathway.^61^ Furthermore, baicalein significantly increased the number of p-nuclear factor erythroid 2-related factor 2 (Nrf2)-positive cells in periodontal tissue and alleviated alveolar bone loss in rats, suggesting a new therapeutic regimen for chronic periodontitis in patients with diabetes mellitus (CPDM).^62^ Curcumin is a polyphenol extracted from curcumin and other plants that has been shown to have anti-inflammatory effects. Curcumin can inhibit NF-κB activation and decrease the OPG/sRANKL ratio in gingival fibroblasts induced by lipopolysaccharide (LPS).^63^ Curcumin promotes the osteogenic differentiation of hPDLSCs via the activation of early growth response gene 1 (EGR1) to accelerate periodontal regeneration.^64^ Because of the instability and toxicity of curcumin, its application is limited. Deng et al. synthesized monocarbonyl analogs of curcumin 1A, which function as host response regulators with increased stability and low toxicity via the nuclear translocation of Nrf2 and the induction of heme oxygenase-1 (HO-1).^65^ A novel chemically modified curcumin 2.24 (CMC2.24) effectively reduced the excessive accumulation of macrophages and damage to chemotactic activity to rescue bone resorption in patients with diabetic periodontitis, indicating that it is an effective treatment for refractory periodontitis.^66^ Duan et al. developed a dual antibacterial hydrogel (CS-PA) scaffold featuring curcumin loaded into biodegradable nanoparticles (CNPs) that was confirmed to enhance the antioxidant capacity of macrophages via the glutathione metabolic pathway, achieving therapeutic effects in periodontitis and hypertension comorbidity mice.^67^ Epigallocatechin-3-gallate (EGCG), a polyphenol extract from green tea, alleviated *Pg*.-induced periodontitis by decreasing the expression levels of inflammatory factors.^68^ A randomized controlled trial further confirmed that an EGCG solution had an additional benefit on the bleeding index and resulted in better outcomes in nonsurgical periodontal treatment, which was overcome by the addition of light and oxygen.^69,70^ Tanshinone IIA (TSA) could inhibit the NF-κB signaling pathway to reduce oxidative stress and *Pg*.-induced atherosclerosis symptoms.^71^ These findings demonstrate that scholars have made great contributions to the promotion and application of natural medicine in clinical practice.

### Periodontal homeostasis

Many studies have shown that osteoblasts and osteoclasts play indispensable roles in maintaining periodontal homeostasis.^72–77^ With the continuous development of science and technology, periodontitis-related research methods are also constantly improving, which broadens the research field for the study of the pathological mechanism of periodontitis. Single-cell RNA sequencing (scRNA-seq) performed to analyze specific cell populations to comprehensively analyze changes in the bone immunological microenvironment in periodontal tissue.^51,78^ Fibroblast clusters constitute the largest number and varieties of cell types identified via scRNA-seq in human periodontal tissue. Mesenchymal stem cells (MSCs) have a fibroblast-like morphology and can differentiate into a variety of cell types, which could characterize the differentiation pathways of osteoblasts and the functional differences in preosteoblast subpopulations via UMAP analyses.^78^ Zhang et al. reported that the percentage of CD301B^+^ macrophages was elevated during the recovery period, whereas the percentage was relatively decreased during the inflammatory period in periodontal tissues. This pathway is located mainly at the beginning of bone repair and promotes osteoblast differentiation.^79^ Jiang et al. confirmed that bromodomain-containing protein 9 (BRD9) interacted with the transcription factor FOXP1 to activate STAT1 transcription and IFN-β signal transduction, thereby affecting the functional changes of osteoclasts. A local drug delivery system containing BRD9 effectively reduced acute bone loss in periodontitis.^80^ However, apart from the oft-studied osteoblasts and osteoclasts, we are still unable to explain the mechanism triggering bone remodeling and guiding the localization of osteoclasts and osteoblasts in periodontitis. Herein, these scientific questions need to be clarified. Li et al. reported that diabetic periodontitis caused alveolar osteocyte ferroptosis by destroying the SLC7A11/GPX4 axis.^81^ The accumulation of prematurely senescent and apoptotic osteocytes was observed in periodontal alveolar bone. Changes in the amount of protein secreted by osteocytes could affect the balance of bone resorption and formation, which may exacerbate local tissue damage.^82^ An increasing number of studies on the pathological mechanism of periodontitis are being conducted in China to comprehensively interpret periodontal diseases and explore therapeutic targets.

### Periodontal ligament stem cells

Periodontal ligament stem cells (PDLSCs) are a unique type of MSCs present in the periodontal ligament and possess multifunctional differentiation potential; these cells are considered important seed cells in periodontal tissue regeneration. Impaired PDLSCs destroy the periodontal microenvironment and aggravate the development of periodontitis.^83^ In the microenvironment of periodontitis, the osteogenic potential of PDLSCs decreases significantly, which might be related to the axis of miR-148a/NRP1.^84^ Sinensetin can reduce Bach1 levels in PDLSCs in a dose-dependent manner and significantly increase the level of the antioxidant stress factor HO-1 in the inflammatory microenvironment.^85^ Low-intensity pulsed ultrasound (LIPUS) is considered an effective method for enhancing the osteogenic differentiation of patient PDLSCs via the suppression of inflammation through the unfolded protein response pathway.^86^ Alveolar bone osteocytes negatively regulate the activity of Gli1^+^PDLSCs through sclerostin, which attenuates PDLSC osteogenic differentiation lineage and delays the renewal of periodontally supported tissue.^87^ Su et al. reported that exosomes from healthy PDLSCs could restore the osteogenic ability of endogenous stem cells in an inflammatory environment and promote the regeneration of alveolar bone.^88^ PDLSC-derived extracellular vesicles (EVs) can significantly promote the osteogenic differentiation and proliferation of MC3T3-E1 cells.^89^ A study revealed that human bone marrow stem cell (hBMSC)-derived EV-miR-1246 could inhibit the expression of angiotensin-converting enzyme 2 (ACE2) and increase the p-Yes-associated protein (YAP)1/YAP1 ratio in CD4^+^ T cells, thus reversing the Th17/Treg ratio in CD4^+^ T cells in periodontitis patients, which might provide a target for periodontitis treatment.^90^ Li et al. demonstrated that the overexpression of secreted crimp-2 in human apical papilla-derived stem cells (SCAPs) can increase the ability to promote tissue regeneration in the periodontal defect area of miniature pigs.^91^ Therefore, further studies are needed to elucidate the function of PDLSCs and provide new treatment plans for periodontitis.

### Periodontal disease and systemic disease

The new classification of periodontal disease also increases risk factor assessment, which is essential for the diagnosis and treatment of periodontal disease. Several risk factors have attracted our attention. The mechanism underlying the association between periodontitis status and systemic disease risk is widely considered to involve the following three common steps: 1. direct diffusion of infection; 2. bacteria entering the blood circulation; and 3. periodontal pathogenic bacteria and their products causing inflammation or an immune response in the body. Infection with *Pg*., a major pathogen of periodontitis, can aggravate neuroinflammation and cognitive dysfunction in Alzheimer’s disease patients by decreasing the proportion of monocytic myeloid-derived suppressor cells (mMDSCs), whereas exogenous supplementation with mMDSCs can restore immune homeostasis and improve the cognitive impairment induced by neuroinflammation and *Pg*. infection. The destruction of periodontal homeostasis can lead to increased and abnormal activation of astrocytes in the hippocampi of mice.^92,93^ Yan et al. reported that periodontitis significantly increased the number of osteoclasts in long bones in mice, changed the composition of the intestinal microbiota and increased the expression of inflammatory markers. The degree of periodontitis is positively correlated with the imbalance of the intestinal flora, suggesting that periodontitis can affect body health through bacteria and other mechanisms.^94,95^ Man et al. reported that inhibiting Rgs10 promoted the progression of periodontitis in mice with periodontitis and rheumatoid arthritis, suggesting that Rgs10 has a potential therapeutic role in periodontitis and rheumatoid arthritis.^96^ Wang et al. reported that nuclear paraspeckle assembly transcript 1 (NEAT1) reduces autophagy flux via the downregulation of syntaxin 17 (STX17), which is considered a target for the production of nicotine-treated PDLSC inflammatory cytokines.^97^ Maintaining good periodontal health in patients with chronic bronchitis and emphysema may help prevent the onset of COPD, according to the results in a large-scale prospective UK Biobank cohort.^98^ Shao et al. summarized various virulence factors derived from periodontitis, regulated islet beta-cell dysfunction and induced peripheral target tissue insulin resistance, which provided new therapeutic ideas for the future implementation of targeted interventions involving virulence factors and cell-specific immunotherapy to treat periodontitis and diabetes in combination.^99^ Scholars have reported that excessive accumulation and delayed removal of neutrophils in the gingival tissue of patients with diabetes can lead to the formation of many NETs, thus aggravating inflammation in patients with diabetes and delaying the resolution of periodontitis-related inflammation.^100^

### Periodontal regeneration

At present, in addition to initial periodontal therapy, such as subgingival scaling and root planning, many patients eventually need periodontal surgery. In recent years, surgical treatments for periodontitis have achieved good results in China with the support of the NSFC. According to the new classification of periodontal and peri-implant diseases based on medical evidence, the stages of periodontitis have been refined.^101,102^ This will make it easier to guide clinicians in choosing more appropriate disease prevention and treatment measures. The updated diagnostic criteria and operational guidelines for periodontitis will greatly guide the diagnosis and treatment of periodontitis. In recent years, a new drug clinical trial of an independently developed “human umbilical cord MSC diaphragm” product has been approved by the Drug Review Center of the State Drug Administration, allowing clinical trials to be carried out in China.^103^ Many experimental studies have confirmed that electrospinning materials can promote functional periodontal tissue regeneration by loading a variety of drugs, bioactive substances and nanoparticles in vivo and in vitro.^104–106^ Cobalt oxide-supported Ir (CoO-Ir) can effectively combat periodontitis by protecting cells from ROS attack, thereby inhibiting inflammatory-induced tissue destruction and promoting osteogenic regeneration^107^. These results provide a preliminary basis for future clinical transformation. In addition, the health and aesthetics of soft periodontal tissue have attracted increasing attention in recent years. The periodontal phenotype plays an important role in the long-term stability of periodontal tissue. Acellular dermal matrix (ADM) has been developed as an alternative to subepithelial connective tissue grafts for the adjuvant treatment of periodontal soft tissue incremental surgery. Lin et al. reported that ADM can promote the migration, adhesion and proliferation of periodontal membrane cells and human oral fibroblasts. Human gingival fibroblasts have been inoculated with human ADM or porcine ADM.^108^ Song et al. used tunnel technology combined with subepithelial connective tissue transplantation to treat lingual gingival retraction of lower anterior teeth, reducing dentin sensitivity and root canal risk for patients.^109^ Considering the complexity of the periodontal environment, Ge et al. synthesized an intelligent gingival reactive hydrogel (PEGPD@SDF-1), which could achieve periodontal bone regeneration in situ in rats via the combined application of a hydrogel, dithiothreitol (DTT) and stromal cell-derived factor-1 (SDF-1).^110^ These Chinese studies indicate that our research is focused not only on the mechanism but also on the clinical manifestations and transformation of periodontitis.

### Co-occurrence analysis of periodontitis research

**Fig. 5:**
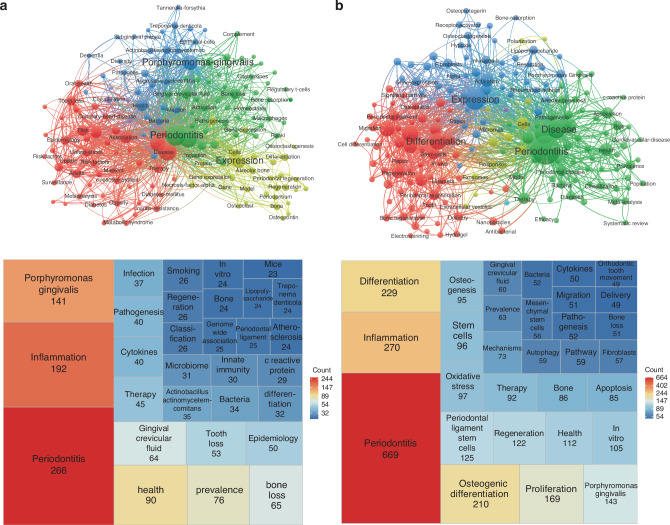
The distribution of the top 30 keywords in the periodontitis related publications supported by the NIH or NSFC from 2014 to 2023. **a** The distribution of the top 30 keywords in the NIH funded periodontitis related publications. **b** The distribution of the top 30 keywords in the NSFC funded periodontitis related publications

The relationship between periodontitis and other related research has been further explored through co-occurrence analysis, which is an effective statistical method for multidimensionally revealing periodontitis. Cooccurrence analysis uses the resource pool of existing publications for further integration analysis, which is highly important for analyzing the research direction and depth of disease detection. Herein, we analyze the keywords that are defined more than 10 times in the publications by using VOSviewer. The 158 keywords were identified from NIH studies and classified into 5 groups: first, the bacterial mechanism is represented by a yellow cluster; second, the clinically relevant features and prognostic analysis are represented by a red cluster; third, the treatment relevant features mechanisms are represented by a blue cluster; and fourth, the immune-related mechanisms are represented by a green cluster. The purple cluster is associated mainly with classification related research (Fig. 5a). As demonstrated in Fig. 5B, 288 keywords related to NSFC research, including inflammation, differentiation, periodontitis, proliferation and osteogenic differentiation, captured the top 5 terms, which covered a wide range of bacteria, regeneration, immune and therapies (Fig. 5b). Considering the frequencies of the top 30 keywords, whenever the studies were sponsored by the NIH or NSFC, scholars paid more attention to periodontitis-related pathogenesis and tissue regeneration. These results indicate that the NIH and NSFC support fundamental research and translational research, which can be used both to explain the occurrence of disease and to treat disease. Overall, we need to utilize more advanced technologies and more novel directions for in-depth analysis of periodontal diseases to obtain innovative results in the future.

## Future perspectives

The field of periodontitis has become a complex and impactful research area involving the integration of multidisciplinary knowledge. Since the policy of reform and opening up in China, vigorous economic growth has greatly promoted the progress of scientific research on periodontitis. Six departments and 16 codes of findings are involved in periodontitis research at the NSFC, indicating that periodontitis research is multidisciplinary. By promoting the development of new drugs, target exploration, the construction of new materials, and clinical transformation, traditional methods of clinical treatment can be improved to advance the prevention and treatment of periodontitis. Tissue engineering studies provide a theoretical cornerstone for preclinical and clinical trials of periodontitis both in vitro and in vivo. Epidemiological studies have identified a variety of diseases as important risk factors for periodontitis. Research on the crucial factors that induce this disease is helpful for further revealing the pathogenesis of periodontitis. For more intensive research on periodontitis, which ranks sixth in the global disease burden, with more than 1 billion people worldwide suffering from the condition, increasing research funding is important for promoting disease research. In the future, the NSFC may promote vigorous development of this research field by supporting the exploration of disease and systemic disease associations and the development and application of innovative technologies and clinical translational materials.

By analyzing the research results of the NSFC’s funding intensity and funding project trends in recent years, the future trends of such research can be identified. Basic research, which accounts for the vast majority of studies, provides rich knowledge for exploring periodontitis. The rich theoretical basis is convenient for later conversion into clinical trials. Several researchers, funded by the NSFC, have begun clinical trials of periodontitis. However, few periodontitis-related clinical trials exist, indicating that clinical trials are still an emerging field of periodontitis research. There is still a long distance between existing basic research and inspiration from basic research to implement the findings in clinical practice. Therefore, translating the findings of basic scientific research into clinical research is an important funding direction for the NSFC in the future. With the improvement of our knowledge of periodontitis, risk factors are continuously being identified. With further interpretation of the disease correlation, the levels of peroxides, glycosylated end products and lipid metabolites produced by diabetes are closely related to the development of periodontitis.^111–115^ The authors used single-nucleotide polymorphisms (SNPSs) to conduct Mendelian randomization analysis and clinical trials, which provided powerful evidence for the causal relationship between periodontitis and hypertension.^116^ Multiple biotechnologies have been integrated to explain the characteristics of periodontitis disease. Periodontal tissues were collected from healthy and periodontal patients. The cells were stratified into three functionally distinct subgroups by single-cell RNA sequencing, fluorescence in situ hybridization, spatial transcriptomics, and multi-omics integration methods to analyze the related markers. These results provided a research basis for the application of anti-CXCL8 monoclonal antibody-targeted therapy and the development of S100A8 gingival crevicular fluid chips in periodontitis.^117^ Obesity and periodontitis have a significant bidirectional relationship. Studies have shown that increased secretion of tumor necrosis factor-α in gingival crevicular fluid in patients with obesity accelerates the progression of periodontitis. However, the underlying mechanism is obscure, and a high body mass index is an important risk factor for the development of periodontitis.^118–120^ Numerous clinical trials, systematic reviews, meta-analyses, and epidemiological studies have demonstrated a high degree of positive correlation between smoking and the incidence, progression, and severity of periodontitis.^121–123^ Previous studies have confirmed that the activation of aryl hydrocarbon receptors, variations in the microbial composition of oral biofilms and impaired neutrophil function might be the mechanisms of smoke-mediated periodontitis; however, the typical mechanism is still in veil.^124–126^ The research consensus on physical fitness is that it is beneficial to guide people to maintain good habits in daily life to increase resistance to periodontitis. In recent years, many studies have shown that changing the oral periodontal flora through the application of probiotics is highly important for personalized periodontal treatment.^127^ The intestinal probiotic AKK (Akkermansia muciniphila (AKK)) and its specific protein Amuc_1100 can colonize the oral mucosa to modulate inflammation and promote the polarization of macrophages toward the M2 phenotype, which could effectively alleviate periodontitis induced by *Pg*. and reduce bone loss.^128^ A database statistical analysis indicates that the intake of probiotics can alleviate periodontal inflammation.^129^ The development of probiotics could be a new direction for periodontal disease treatment.

With the innovation of science and technology, different types of bioinformatics data analysis continue to emerge, providing new possibilities for the study of periodontitis. The NSFC focused on the development of cutting-edge technologies and provided substantial support and promotion for periodontitis research in China. The specificity and heterogeneity of different cell types in periodontitis can be explained through scRNA-seq mapping, which provides a potential mechanism for exploring specific cell subtypes and cell properties.^130^ Stereo-seq was used to map the high-resolution spatial transcriptome of gingival tissue. Compared with those in healthy individuals, gingival epithelial cells can be divided into 3 subgroups; moreover, the abundance of plasma cells and T cells in gingival tissues significantly increased, and the number of basal layer cells in inflammatory gingival tissues increased. Further analysis revealed that the TGFβ, JAK-STAT and NF-κB signaling pathways were most strongly activated in the endothelial cells of inflammatory tissues. This is highly important for the subsequent screening of disease targets.^51^ Single-cell transcriptome sequencing analysis revealed the temporal and spatial dynamics of gene expression, population composition, and fine interactions during the progression of periodontitis in the bone immune system. Fibroblasts in periodontitis tissues are heterogeneous and can be divided into four groups: 6 fibroblast clusters, 2 pericyte clusters, 1 myofibroblast cluster and a group of proliferating cells. Fibroblast clusters can be further divided into four subclusters related to fibroblasts with unique phenotypes and functions and three osteoblast lineage cell subpopulations, namely, CD55+ MSCs, APOE^+^ pre^-^OBs and IBSP^+^ OBs. Gli1^+^ PDLSCs were found to markedly induce rapid neutrophil migration and aberrant activation. Mechanistically, Gli1^+^ PDLSCs derived EVs enhanced neutrophil ROS generation and stimulated NF-κB signaling activation,which provided novel mechanistic insights into the pathogenesis of periodontitis.^131^ These findings provide insights into the cellular and molecular basis of periodontal bone regeneration.^78^ Through scRNA-seq analysis, Zhang et al. identified CD301b^+^ macrophages as pivotal regulators of the osteoimmunological microenvironment. The research demonstrated that Tim4 orchestrates macrophage polarization via MAPK signaling activation in periodontitis, thereby proposing a macrophage-centric therapeutic paradigm for treatment of periodontitis.^132^

Owing to the collaborative progress of imaging and computer technologies, the applications of artificial intelligence and digital medicine have expanded significantly, playing vital roles in disease risk assessment, detection, diagnosis, prognosis, and other related aspects.^133,134^ The precise localization and removal of dental plaque hold immense importance in the management of periodontal diseases and in guiding patients in maintaining oral hygiene. An automatic dental plaque detection technology based on deep learning has been developed that uses deep neural networks to extract and recognize oral images, enabling the automatic identification and localization of dental plaque. This technology enhances the precision and efficiency of dental plaque detection, offering novel tools and approaches for oral health management and treatment.^135^ A deep learning (DL) model based on computer-aided diagnosis (CAD) was developed to measure the radiographic alveolar bone level to aid in periodontal diagnosis and exhibited high accuracy and high efficiency in making personalized treatment plans and improving the effect of periodontal disease treatment.^136^ Su et al. presented an AI-driven method for automatic periodontal ligament (PDL) segmentation in cone-beam computed tomography (CBCT) images, enabling real-time measurements to aid periodontists, orthodontists, prosthodontists, and implantologists in more accurate and efficient treatment planning.^137^

Increasing the number of teeth that are retained in the oral cavity is highly beneficial for improving the quality of life of patients with periodontitis. In the clinic, the successful treatment of periodontitis is defined as a decrease in the depth of the periodontal pocket, alleviated inflammation (bleeding, redness and swelling of the gums) and controllable oral hygiene maintenance. Ideally, the improvement of clinical symptoms should also be accompanied by increases in clinical adhesion (CAL) and bone mass. Sufficient evidence exists that, in most cases, these goals can be achieved through the first and second steps of periodontal treatment. The first step includes risk factors related to patients with plaque biofilms and controlling periodic diseases. The second step involves fixing nonsurgical gingival equipment.^101,138^ Therefore, there is an urgent need to increase investment in tissue engineering research and promote periodontal bone regeneration for the clinical treatment of periodontitis. In recent years, great progress has been made in stem cell therapy for the clinical treatment of periodontitis. The use of stem cell membranes in the treatment of periodontitis has been proven to significantly improve the therapeutic effect on periodontitis patients and provides help for large-scale translational potential in the later stage in China.^139,140^ The application of stem cells in the treatment of periodontitis is promising worldwide. Research involving tissue engineering experiments includes the continuous accumulation of a series of research results, from the functional maintenance and scale expansion of seed cells and the generation of scaffold materials to application verification in large animal model studies and clinical regeneration, which provide a guarantee for the ultimate realization of periodontal tissue regeneration. For example, the use of EV-coated cell aggregates that promote periodontal tissue regeneration may be an attractive therapeutic approach in the future. 3D-printed poly(ε-caprolactone) (PCL) materials modified with graphene oxide (GO) have been shown to increase the osteogenic differentiation ability of PDLSCs.^141^ S Song et al. reported that LIPUS stimulation of EVs produced by human dental pulp MSCs (SCAPs) can improve the ability of these vesicles to promote bone formation and reduce inflammation.^142^ Vesicles of two block copolymers with different functions have been designed to efficiently deliver low doses of antibiotics to biofilms to treat periodontitis.^59^ The regeneration of periodontal tissue complexes was realized successively in small and large animal models via dental stem cell (DSC) self-assembly polymerization technology. In the process of analyzing the mechanism of regeneration, three essential stages of tissue regeneration that can be used for regeneration applications were proposed: assembly, patterning and crosstalk. Under the guidance of preclinical regeneration practice experience, clinical trials have been carried out, and innovative results have been achieved. In the process of bone regeneration in individuals with periodontitis, local tissue inflammation has adverse effects on the regenerative potential of cell aggregates. Regeneration efficiency should be optimized by preconditioning DSCs or remodeling the host microenvironment.^143^ In recent years, scholars have reviewed the application prospects of vesicles in periodontitis in detail, suggesting that this treatment is being widely promoted in the future.^144,145^ The existing therapeutic regimens rely mainly on initial periodontal therapy. There is no similar noninvasive periodontal tissue regeneration guide technology for local injection treatment, and at present, there are no similar products worldwide. To address the problem of low stem cell sources, researchers have shown that the application of allogeneic exfoliated deciduous teeth (SHEDs) could effectively repair periodontal tissue defects in a swine model.^146^ Wang et al. reported that local injection of hepatocyte growth factor (HGF) and human dental pulp stem cells (DPSCs) promoted periodontal tissue regeneration in a swine model.^147^ When human dental pulp stem cells (hDPSCs) are transplanted into periodontal bone defects, bone regeneration can occur whenever hDPSC injection or cell sheets are used.^148^ The development of human dental pulp mesenchymal stem cell injection products has led to the initiation of clinical trials, which will provide breakthrough results from fundamental research to the clinical application of periodontal regeneration in China. With the rapid development of experimental equipment and biotechnology and the promotion of interdisciplinary combined treatment concepts, the treatment strategy for periodontitis has continuously improved. The NSFC has been committed to promoting interdisciplinary research.

With the increasing trend of population aging and improvements in people’s quality of life, periodontitis-related facial changes and loss of chewing ability have resulted in a substantial economic burden on families and society. Therefore, the importance and urgency of periodontitis disease treatment are becoming increasingly important. The development of new drugs based on research into the mechanism of periodontitis contributes to improving the effectiveness of periodontitis treatments in the clinic. Since the establishment of the Health Science Department of the NSFC in China, the research scope and research strategies involved in the field of periodontitis have increased, as has number of associated researchers, which is very important for promoting the development of research in the field of periodontitis in China. However, there is still a wide gap between fundamental research on periodontitis in China and that in developed countries. The United States has invested numerous funds and advanced scientific research equipment in periodontitis research and possesses an international advanced research platform that provides the necessary guarantee for the output and transformation of its achievements in the field of scientific research. Periodontal tissue destruction is associated with increased complement activity, which can be easily detected in the gingival and gingival crevicular fluid (GCF) in periodontitis. AMY-101 is a complement C3-targeted therapy that blocks downstream pathways in the complement activation cascade, which has been verified in clinical trials.^149^ This development provides new evidence for the local administration of medicines to combat periodontitis in the future.^150,151^ The United States has consistently led in the development of antibiotics and other pharmaceutical agents. Medications such as metronidazole, chlorhexidine, and minocycline have become common in the clinical management of periodontitis. Additionally, a range of developed products, including Oule B, have secured a significant competitive edge in the market. Periodontal disease is a significant age-associated disorder, and therapeutic strategies aimed at the fundamental biological processes of aging hold great promise for effective treatment. Importantly, rapamycin, an FDA-approved medication, has the capacity to reverse age-related periodontal disease in preclinical mouse models, thereby paving the way for novel pharmacological approaches to managing periodontitis.^152^ Numerous successful examples indicate that China should synthesize findings from basic research endeavors and actively pursue their clinical translation.

Numerous efforts should be made in the periodontitis field to solve bottleneck issues. The research level and platform need to be continuously enhanced. With the support of the NSFC community, Chinese scientists will continue to improve in originality and innovation exploration and will cultivate many research groups and young talents to achieve valuable contributions in the field of periodontitis research. Using these resources, we should capitalize on scientific and technological advancements to explore periodontitis. By integrating single-cell spatiotemporal analysis, we aim to elucidate the intricate interplay among the host, microenvironment, and pathogens, providing a comprehensive understanding of the therapeutic targets for periodontitis. In parallel, we are vigorously advancing ‌clinical trials of innovative gene and microbial therapies‌, striving to accelerate their transition from research to clinical practice. These dual approaches will usher in a transformative era of personalized and precision-driven treatments for this widespread oral condition.

## Data Availability

All data generated for this study are available from the corresponding authors upon reasonable request.
